# Influenza Virus Neuraminidase Structure and Functions

**DOI:** 10.3389/fmicb.2019.00039

**Published:** 2019-01-29

**Authors:** Julie L. McAuley, Brad P. Gilbertson, Sanja Trifkovic, Lorena E. Brown, Jennifer L. McKimm-Breschkin

**Affiliations:** ^1^ Department of Microbiology and Immunology, The University of Melbourne at the Peter Doherty Institute for Infection and Immunity, Melbourne, VIC, Australia; ^2^ Department of Infectious Diseases, St Jude Children’s Research Hospital, Memphis, TN, United States

**Keywords:** influenza, neuraminidase, hemagglutinin, sialic acid, infection, HA:NA balance, NA

## Abstract

With the constant threat of emergence of a novel influenza virus pandemic, there must be continued evaluation of the molecular mechanisms that contribute to virulence. Although the influenza A virus surface glycoprotein neuraminidase (NA) has been studied mainly in the context of its role in viral release from cells, accumulating evidence suggests it plays an important, multifunctional role in virus infection and fitness. This review investigates the various structural features of NA, linking these with functional outcomes in viral replication. The contribution of evolving NA activity to viral attachment, entry and release of virions from infected cells, and maintenance of functional balance with the viral hemagglutinin are also discussed. Greater insight into the role of this important antiviral drug target is warranted.

## Introduction

Influenza viruses are members of the family *Orthomyxoviridae*, a group of enveloped viruses containing a segmented negative-sense single-stranded RNA genome. Three main types of influenza viruses (A, B, and C) infect humans, with influenza A and B viruses causing significant morbidity and mortality annually. While yearly outbreaks of influenza in the human population induce the development of neutralizing antibody against common circulating strains, new strains arise constantly in a process referred to as antigenic drift. This occurs due to errors in copying of the genome by the viral polymerase and selection of those mutants with changes in the antigenic sites that allow escape from neutralization [reviewed in (Wilson and Cox, 1990)].

The influenza virus major surface glycoproteins, hemagglutinin (HA), and neuraminidase (NA) dominate the virion surface and form the main targets for these neutralizing antibodies. In addition to the mutations that arise due to antigenic drift, the HA and NA of influenza A viruses (IAVs) can exist in different forms. Based on HA and NA antigenicity using serologic tests with hyperimmune sera, there have been a total of 16 HA (H1-16) and 9 NA (N1-9) subtypes identified in birds. These are expressed in numerous combinations of viruses isolated from aquatic avian species, and an additional two combinations, H17N10 and H18N11, have been identified in bats (Tong et al., 2012, 2013). IAVs of subtypes H1N1 and H3N2 are endemic in humans, circulating constantly within the population and giving rise to seasonal outbreaks. Zoonotic transmission from birds and swine of viruses with certain other HA and NA combinations (e.g. H5N1, H7N9, and H9N2) sporadically occurs; however, these viruses need to accrue additional mutations in order to gain the ability to transmit readily between humans (Cox and Subbarao, 2000; Harris et al., 2017). If viruses bearing a novel HA subtype, which is often accompanied by a novel NA, gain the ability to transmit between humans, the potential exists to cause another pandemic, as no one will have relevant pre-existing neutralizing antibody immunity to this novel virus.

The functions of both HA and NA involve interaction with sialic acid, a terminal structure bound to underlying sugar residues expressed by glycoproteins or glycolipids at the cell surface (Gottschalk, 1958; Palese et al., 1974). The binding of HA to sialic acids presented by cellular receptors triggers cell entry by clathrin-mediated endocytosis, although other endocytic routes, including micropinocytosis, may also be used [reviewed in (Lakadamyali et al., 2004)]. A major function of NA occurs in the final stage of infection. Viral NA removes sialic acids from both cellular receptors and from newly synthesized HA and NA on nascent virions, which have been sialylated as part of the glycosylation processes within the host cell (Palese et al., 1974; Basak et al., 1985). NA cleavage of sialic acids prevents virion aggregation and stops virus binding back to the dying host cell *via* the HA, enabling efficient release of virion progeny and spread to new cell targets (Palese et al., 1974).

The role of the viral HA in attachment and infection has been well explored, yet examination of the role of NA in the IAV infection cycle has been largely limited to its role in aiding exit of virion progeny from infected cells. The majority of reviews on NA have focused on viral inhibitors that target NA and block this function. While some studies have suggested NA function does not influence the early stage of IAV infection (Liu et al., 1995), arguably, the sialidase activity of NA aids the virus to gain access to cells by catalyzing the cleavage of sialic acids presented by decoy receptors, such as mucins (Kesimer et al., 2009; McAuley et al., 2017), potentially providing NA with an important role in viral entry. In addition, experiments showing a decrease in infection of cells in the presence of NA-blocking drugs provide evidence for a role of NA in a virus entry step (Matrosovich et al., 2004; Ohuchi et al., 2006; Su et al., 2009; Gulati et al., 2013). As such there is a need for better understanding of the complex role of NA in the influenza infection and replication cycle, particularly with consideration to how the disparate roles of HA and NA glycoproteins need to achieve a functional balance in order to maintain viral fitness. Therefore, we sought to review the existing literature to evaluate the NA structure and function in relation to its role in the IAV infection cycle.

## NA Structure

**Figure 1:**
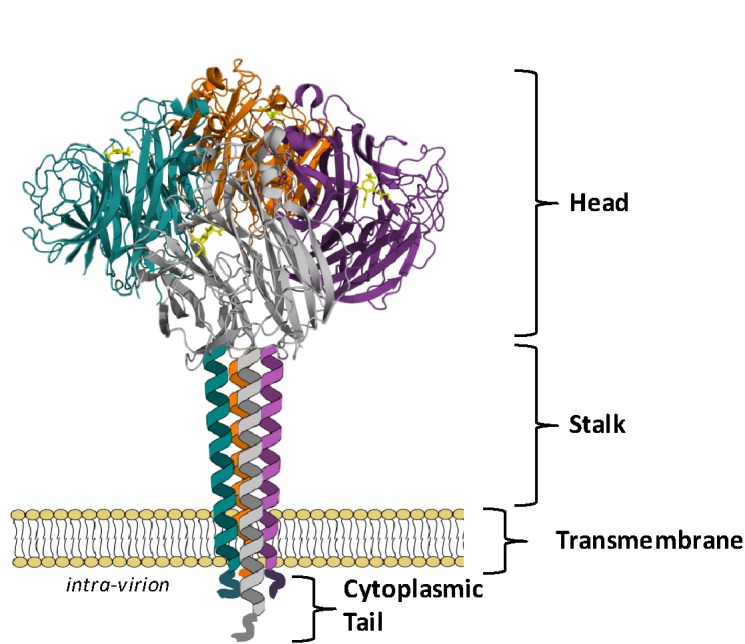
NA exists as a tetramer of four identical monomers. Each monomer consists of four distinct structural domains known as the catalytic head, the stalk, the transmembrane region and the cytoplasmic tail. The head domain structure was generated in Pymol using structural information from Protein Data Bank code 4GZX (A/Tanzania/205/2010 N2 NA). Individual head domain monomers are shown in green, grey, purple, and orange. The NA stalk, transmembrane region, and cytoplasmic tail are yet to be resolved and are depicted here as four alpha helices.

The NA assembles as a tetramer of four identical polypeptides and, when embedded in the envelope of the virus, accounts for approximately 10–20% of the total glycoproteins on the virion surface, with about 40–50 NA spikes and 300–400 HA spikes on an average sized virion of 120 nm (Varghese et al., 1983; Ward et al., 1983; Moules et al., 2010). The four monomers, each of approximately 470 amino acids, fold into four distinct structural domains: the cytoplasmic tail, the transmembrane region, the stalk, and the catalytic head (Figure 1). Cryoelectron tomography studies have indicated that the NA tetramer exists in local clusters on the virion surface or as isolated spikes surrounded by HA (Harris et al., 2006). Depending on the length of the stalk region, the NA may protrude slightly more (Harris et al., 2006) or less (Matsuoka et al., 2009) above the viral envelope than the HA, which may influence the overall enzymatic activity of the virus.

### Cytoplasmic Tail

Suggesting that the NA cytoplasmic tail is involved in critical viral functions, the N-terminal domain sequence is nearly 100% conserved across all IAV subtypes and consists of the sequence MNPNQK (Blok and Air, 1982). Reverse engineered viruses containing site-specific mutations in this domain exhibit altered virion morphology and reduced replicative yields (Mitnaul et al., 1996; Jin et al., 1997; Barman et al., 2004). IAV engineered to encode an NA lacking a cytoplasmic tail could still be rescued albeit with a markedly attenuated phenotype (Garcia-Sastre and Palese, 1995). The altered morphology and attenuated infectivity of viruses expressing NA lacking the cytoplasmic tail domain are thought to be due to a lack of interaction with the membrane-associated matrix M1 viral protein (Enami and Enami, 1996), which ultimately alters the efficiency of budding from the infected host cell (Jin et al., 1997; Ali et al., 2000; Barman et al., 2001; Mintaev et al., 2014). Determinants in both the cytoplasmic tail domain and the transmembrane domain contribute to the transport of the glycoprotein to the apical plasma membrane (Kundu and Nayak, 1994; Kundu et al., 1996). However, the role of the tail domain in packaging the surface NA into virions remains unclear. A complete loss of the tail domain (Garcia-Sastre and Palese, 1995) resulted in a 50% reduction in the amount of NA in infected cells. This corresponded to a reduction in the amount of NA incorporated into virions, suggesting efficient packaging of existing NA. However, the absence of all tail amino acids except for the initiating methionine gave rise to virus that also showed markedly less incorporation of NA into virions, but in this case, NA was present at the plasma membrane at similar levels to wild-type virus (Mitnaul et al., 1996).

### Transmembrane Domain

The N-terminal hydrophobic transmembrane domain, which attaches the NA to the viral envelope (Bos et al., 1984), contains a variable sequence of amino acids spanning residue numbers 7–29 and is predicted to form an alpha helix (Blok and Air, 1982; Air, 2012) with interspersed polar residues driving subunit-subunit interactions (Nordholm et al., 2013). The transmembrane domain provides signals for translocation from the endoplasmic reticulum to the apical surface, as well as association with lipid rafts (Barman and Nayak, 2000). The N-terminal amino acids (positions 1–74), which include both the cytoplasmic tail, the transmembrane domain, and some of the stalk region have been reported to be sufficient to target the cell membrane and for the formation of the NA tetramer complex (Kundu et al., 1991, 1996; da Silva et al., 2013; Nordholm et al., 2013). Implicating the function of the transmembrane domain directly in the translocation of NA to the apical membrane, membrane trafficking can also occur in the absence of the stalk and head domain (Ernst et al., 2013). Mutation of specific amino acids within the transmembrane domain can induce stable architectural differences in the anchoring signal region that result in diminished transport to the plasma membrane (Ernst et al., 2013).

### Stalk

The stalk domains of NAs of different IAV subtypes share some structural features, but the number and sequence of amino acid residues can vary considerably (Blok and Air, 1982). Regardless of this variability, all NA stalk domains share some structural features, including at least one cysteine residue and a potential glycosylation site (Blok and Air, 1982; Air, 2012). The cysteine residue(s) may assist with tetramer stabilization by enabling disulfide bonds to form between each monomeric NA unit (Blok and Air, 1982; Ward et al., 1983). While the cysteine residues may occur at variable positions in the NA stalks of different subtypes, the tetrameric structure of NA allows them to align, so that the disulfide bonds can form between the pairs of cysteine residues on neighboring monomers (Blok and Air, 1982). The presence of carbohydrate side chains within the stalk is thought to contribute further to the stability of the tetramer (Blok and Air, 1982).

**Figure 2:**
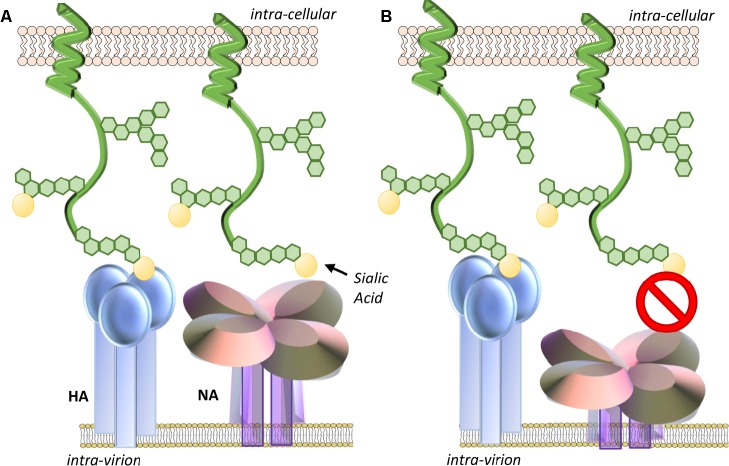
**(A)** Optimal NA stalk length enables the NA catalytic head access to sialic acid-expressing receptors. **(B)** Reduced stalk length may impact the ability of NA to contact sialic acids on mucins or cellular receptors as neighboring HA may sterically hinder its approach. The degree of impact may be dependent on the ratio and spacing of HA and NA glycoproteins on the virion surface. Reduced stalk size may also shift the geometry and dynamics of the enzymatic pocket altering its affinity for sialic acid.

The length of the stalk region of different subtypes can have significant impact on particular virus characteristics. Using reverse engineering techniques, mutant viruses unable to produce the NA stalk were able to be rescued in tissue culture cells and replicate to the equivalent titer as the unmodified parent virus, but could not replicate in eggs or mice (Castrucci and Kawaoka, 1993). Using a series of NA mutants differing only in stalk length, studies showed that while there was no correlation between the stalk length and the ability to cleave fetuin or a small substrate *in vitro*, enhanced virus replication in eggs correlated closely with increasing stalk length (Els et al., 1985; Castrucci and Kawaoka, 1993). Viruses presenting NAs with shortened stalk domains have also been reported to elute less efficiently from chicken erythrocytes (Els et al., 1985; Castrucci and Kawaoka, 1993). Reduced stalk length has been commonly thought to impact NA activity of virions because the diminished height may hinder access to cellular sialic acid expressing receptors and that the towering HA blocks the shorter NA catalytic domain from gaining access to the sialic acids (Baigent and McCauley, 2001) (Figure 2).

More recently, it has been postulated that the limited access to substrate theory may not fully explain the reduced sialidase activity of stalk-deletion mutants, with the argument that the clustering of NAs on the virion surface would diminish any blocking effects of nearby HA. This view was backed up by molecular dynamics simulations providing evidence that a reduced stalk size also alters the geometry and dynamics of the enzymatic pocket itself, modifying its affinity for sialic acids (Durrant et al., 2016). Further evidence for the impact of the stalk structure on the activity of the NA catalytic domain comes from experiments where the same NA head domain was expressed with different tetramerizing domains as artificial stalks (Schmidt et al., 2011). A tetrabrachion stalk conferred similar properties as the native NA, whereas the yeast stalk (GCN4-pLI) reduced substrate affinity and inhibitor binding. Others have also demonstrated that a single mutation in the stalk can significantly affect enzyme activity, presumably affecting the stability of the tetramer (Zanin et al., 2017).

There is also a mounting evidence for a relationship between NA stalk length and virulence in mammalian models of infection. NA stalk truncation mutants of the 2009 pandemic virus A(H1N1)pdm09 showed greater lethality in mice and virulence in ferrets than the untruncated counterpart (Park et al., 2017). Loss of the glycosylation site in the stalk likewise enhanced virulence in mice (Park et al., 2017). A deletion of 20 amino acids is frequently observed upon transmission of IAV from waterfowl to domestic poultry (Matsuoka et al., 2009; Hoffmann et al., 2012; Blumenkrantz et al., 2013; Sun et al., 2013; Bi et al., 2015). As such, the length of the stalk domain may be a species-specific determinant of viral adaptation and virulence that we are yet to fully understand (Li et al., 2014; Stech et al., 2015; Park et al., 2017).

### Head Domain

**Figure 3:**
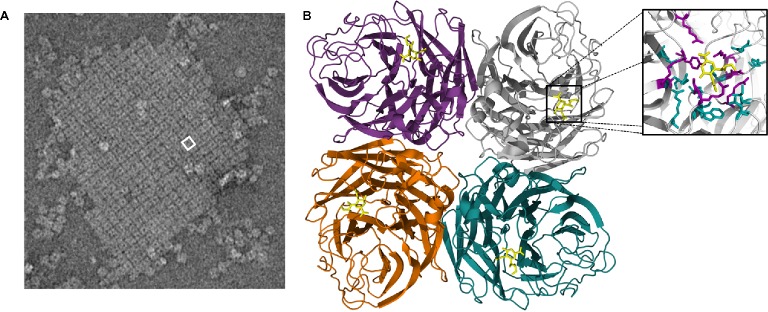
**(A)** An electron micrograph of a two-dimensional crystal array formed by NA heads, generated as described by Oakley et al. (2010). One square-shaped structure = one tetramer head, with the hole in the center of each tetramer. White box represents one tetramer head, which is depicted in the structural cartoon in B. **(B)** The catalytic head of NA consists of a six-bladed propeller structure, with each blade of the propeller having four anti-parallel β-sheets that are stabilized by disulfide bonds and connected by loops of variable length. Sialic acid (yellow structure) is observable on the head of each monomer. The boxed region is magnified in the inset and shows the receptor-binding pocket to which sialic acid (yellow compound) binds. The structure was generated in Pymol using structural information from Protein Data Bank code 4GZX.

Peptide maps from crystallized NA catalytic heads were first detailed in 1978 (Laver, 1978). However, the actual structure of this domain was first described in 1983 for IAVs and in 1992 for IBVs (Varghese et al., 1983; Burmeister et al., 1992). The catalytic head of all NAs consists of a box-shaped structure comprising four monomers (Figure 3). Each monomer is in the form of a six-bladed propeller structure, with each blade having four anti-parallel β-sheets that are stabilized by disulfide bonds and connected by loops of variable length (Varghese et al., 1983). A functional catalytic site is present on the surface of each monomer and is directed sideward rather than upward, a property consistent with the ability to cleave sialic acids from nearby membrane glycoproteins to prevent virus trapping (Colman et al., 1983; Burmeister et al., 1992). These catalytic sites are characterized by a large cavity with an unusually large number of charged residues in the pocket and around its rim (Colman et al., 1983; Varghese et al., 1992). The tetrameric form of NA is considered optimal for enzyme activity, and mutations that lead to instability of the tetramer lead to decreased enzyme activity (McKimm-Breschkin et al., 1996b; Fujisaki et al., 2012; McKimm-Breschkin et al., 2013). While it has been reported that monomers alone have no enzyme activity (Air, 2012) and usually expression of recombinant soluble NA heads requires a synthetic tetramerization domain for active NA (Schmidt et al., 2011), there are reports of expression of soluble recombinant monomeric influenza NA heads in both yeast and mammalian cells that have comparable properties to the native enzyme (Yongkiettrakul et al., 2009; Nivitchanyong et al., 2011). When the head domain of NA is proteolytically cleaved from the remaining NA tetrameric stalk embedded in the virion, the enzymatic properties remain active and the heads retain the tetrameric state of purification (Laver, 1978; McKimm-Breschkin et al., 1991).

**Figure 4:**
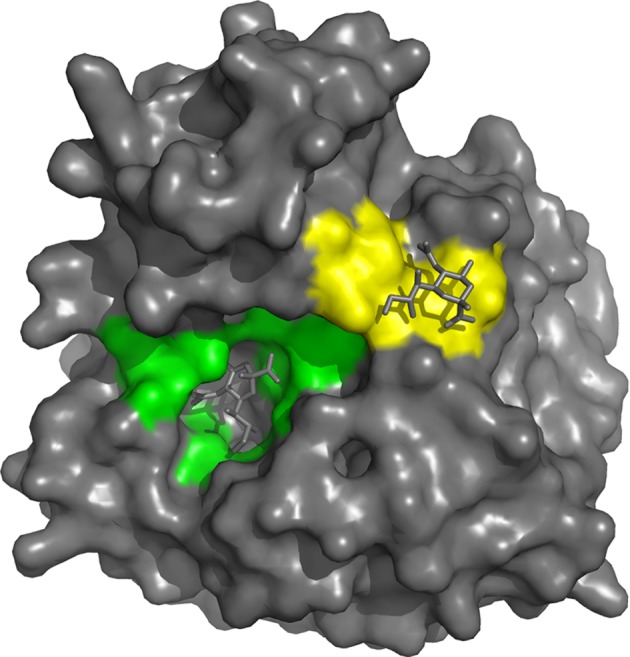
On the basis of interaction with sialic acid, residues Arg118, Asp151, Arg152, Arg224, Glu276, Arg292, Arg371 and Tyr406 are considered as the catalytic sites that mediate cleavage from the underlying sugar residues presented by glycoproteins and are highlighted in green. The second site binds sialic acid by making contacts with Ser367, Ser370, Ser372, Asn400, Trp403 and Lys432 (highlighted in yellow), but bound sialic acid at this site is not released by the NA activity. The structure was generated in Pymol using structural information from Protein Data Bank code 1MWE for the A/tern/Australia/G70C/75 NA/Neu5Ac complexed at 4 °C.

The NA active site consists of an inner shell of eight highly conserved residues that interact directly with sialic acids (Arg118, Asp151, Arg152, Arg224, Glu276, Arg292, Arg371, and Tyr406) (Figure 4). In addition, there is an outer shell of 10 residues, which do not contact sialic acid, but which have an important structural role and are defined as framework residues. These comprise Glu119, Arg156, Trp178, Ser179, Asp198, Ile222, Glu227, Glu277, Asn294, and Glu425 (Colman et al., 1983, 1993; Burmeister et al., 1992). Three arginine residues (Arg118, 292, 371) interact with the carboxylate of the sialic acid substrate. Arg152 binds to the acetamido group on the sugar ring, while Glu276 interacts with the 8- and 9-hydroxyl groups on the glycerol side chain. The enzyme active site is said to be highly conserved in both spatial orientation and sequence properties, making it an ideal target for drug inhibition.

NA active site variants that either occur naturally or are created *via* genetic modification have shown that both framework and catalytic residues can alter the viral replicative ability, transmissibility, and susceptibility to antiviral inhibitors to varying degrees (Lentz et al., 1987; McKimm-Breschkin, 2000, 2013; Abed et al., 2006; Meijer et al., 2009; Richard et al., 2011; Chao et al., 2012; Govorkova, 2013). An H1N1 virus that emerged in 2007–2008 had a single H275Y amino acid change conferring oseltamivir resistance (Hurt et al., 2011; Takashita et al., 2015). This virus showed no decrease in fitness, spreading globally over the next year. Therefore, it remains important to monitor antiviral susceptibility of viruses circulating in the community.

## Structural Relationships between NA Subtypes

Phylogenic mapping, which included comparisons of genetic and structural relationships between NAs from different viruses (not including the recently discovered bat viruses) revealed that IAV NAs fall into two distinct groups, regardless of their serotype identification (i.e. N1–N9) (Russell et al., 2006). Group 1 contains N1, N4, N5, and N8 serotypes, while group 2 contains N2, N3, N6, N7, and N9 serotypes. Crystal structures of the group 1 and group 2 NAs reveal a particularly striking feature in the catalytic domain referred to as the ‘150 loop.’ This loop comprises residues 147–152, which forms one corner of the enzyme active site, and is able to exist in at least two conformations (Russell et al., 2006). Group 1 NA structures have an additional cavity next to the active site, which is created by the movement of the 150 loop during conformational changes brought about by substrate binding within the active site. Structurally, the group 2 NAs do not appear to have the cavity. By X-ray crystallography, the A(H1N1)pdm09 NA also appears to lack the 150-cavity and has more structural similarities to the group 2 NAs (Li et al., 2010; Amaro et al., 2011). However, molecular dynamics studies suggest that the A(H1N1)pdm09 NA and the group 2 NAs do have the 150-loop flexibility, but there may be fewer of the population of the open 150-loop conformation, hence not seen in the static structures in crystals (Amaro et al., 2011). As NA inhibitors have been developed based on crystal structures of group 2 NAs, there is now a great interest in the development of novel inhibitors that target the cavity of group 1 NAs (Russell et al., 2006; Li et al., 2010; Amaro et al., 2011). Sialic acid derivatives that selectively lock the 150 loop in the open cavity conformation inhibit the group 1 sialidases, including the A(H1N1)pdm09 NA, further supporting the fact that this pandemic NA does have an accessible 150 cavity that is exposed to some extent upon the binding of an appropriate inhibitor (Rudrawar et al., 2010).

### NA Hemadsorption Site

X-ray crystal structures of N6 and N9 that have revealed sialic acids can also bind at a discrete second site on the NA head (Varghese et al., 1997; Air, 2012; Streltsov et al., 2015), resulting in the binding of red blood cells (hemadsorption) to the NA. The second site, a shallow pocket located next to the catalytic site, is made up of three surface loops and binds sialic acid by making contacts with Ser367, Ser370, Ser372, Asn400, Trp403, and Lys432 (Figure 4), but bound sialic acids at this site are not released by the NA activity (Varghese et al., 1997). The second site appears to be a common feature of avian NAs of different subtypes, while swine influenza strains have lost several of these conserved residues, so theoretically they do not have the second site (Kobasa et al., 1997; Varghese et al., 1997). However, the Brownian dynamics simulation of human N2 from the 1957 pdmIAV and the A(H1N1)pdm09 NA suggests that some of the key structural features are retained (Sung et al., 2010), and NMR studies subsequently added support to the presence of the second site in these viruses (Lai et al., 2012). Substitutions at different positions in the three loops of the second sialic acid-binding site on the NA of human H1N1 IAV have been shown to have a profound effect on binding and cleavage of multivalent, but not monovalent, receptors and significantly affect virus replication (Du et al., 2018). Linking the second sialic acid-binding site with host tropism, it has been reported that for avian viruses that have succeeded in zoonotic transmission to man, such as the pandemic 1918 and 1968 IAVs and the emerging H7N9 IAV, this site contains point mutations (Uhlendorff et al., 2009; Dai et al., 2017). It has been hypothesized that substitutions in the second sialic acid-binding site enable the enzymatic activity of the NA of newly emerging viruses to be compatible with an HA that is evolving toward human receptor-binding specificity (Du et al., 2018).

Understanding the biological function of the NA second sialic acid-binding site has been challenging. One study has shown that the ability of NA to bind red blood cells correlated with the cleavage efficiencies of multivalent substrates like fetuin (Uhlendorff et al., 2009). The observation that substrate binding *via* the second sialic acid-binding site in H7N9 viruses enhances NA catalytic efficiency against the same substrate (Dai et al., 2017) is possibly achieved by recruiting and keeping multivalent sialosides close to the active site (Uhlendorff et al., 2009; Dai et al., 2017). The second site has also been shown to be a highly conserved target for a novel influenza inhibitor found in the saliva of mice that impact the ability of the infection to progress to the lungs of infected mice when the inoculum is initially confined to the upper respiratory tract (Ivinson et al., 2017). The amino acids at positions 368–370 (N2 numbering) on the rim of the second site dictated the *in vitro* susceptibility of different viral strains to the salivary inhibitor and their ability to progress to the lungs of mice (Gilbertson et al., 2017).

## NA Functional Roles in Replication

### Virus Entry

NA activity and cleavage of sialic acids have long been thought to enable movement of the virion through mucus (Burnet, 1948). Airway mucus is comprised of a large network of sialylated glycoproteins known as gel-forming mucins (MUC5AC and MUC5B in particular), which interconnect and serve as a scaffold to present secreted antiproteases, antioxidants, antimicrobial proteins, secretory immunoglobulins (IgA), cytokines, and other innate defensive molecules (Lillehoj and Kim, 2002). Hypersecretion of mucus during the course of respiratory tract infection can occlude the airways and increase the lung pathology (Rose and Voynow, 2006). In murine models of influenza infection, overexpression of the major respiratory tract gel-forming mucin, Muc5ac (non-human form of MUC5AC), revealed that this glycoprotein presents SAα2-3Gal, which can bind virus and limit infection of the underlying epithelia (Ehre et al., 2012). This supports the proposal that the physical properties of the gel-forming mucins serve as a trap by presenting decoy receptors to which the inhaled pathogen binds and is then cleared by way of the mucociliary escalator (Button et al., 2012). IAV has been shown to interact with secreted mucus on frozen human trachea and bronchus tissue sections, and bead-bound mucins inhibited the NA cleavage of substrate (Cohen et al., 2013). To add credence to the hypothesis that NA functions to aid viral movement through the respiratory mucus layer, NA inhibitors have been shown to block IAV entry into differentiated human tracheobronchial and nasal epithelial cells, as well as porcine cells that secrete mucus (Matrosovich et al., 2004; Yang et al., 2014). Exogenous NA also enhanced passage through the mucus layer (Yang et al., 2014). This suggests that NA is needed to remove decoy sialic acids presented on mucins, cilia, and cellular glycocalyx in order for virus to efficiently access functional receptors on the surface of target cells.

### Receptor Binding

In addition to the non-catalytic sialic-binding site that is structurally distinct from the NA active site in N6 and N9 NAs (Varghese et al., 1997; Air, 2012; Streltsov et al., 2015), more recent studies have shown that the NAs of human H3N2 viruses isolated since 1994 can also demonstrate agglutination of red blood cells after passage in MDCK cells, but not in eggs (Lin et al., 2010; Hooper and Bloom, 2013; Mohr et al., 2015). This property of the more recent N2 NAs was first noted because many H3N2 isolates showed weak HA-mediated binding to chicken red blood cells allowing NA-dependent hemagglutination to be detected. NA agglutination is inhibited by NA inhibitor drugs (NAIs) but only poorly inhibited by post-infection ferret antisera, thus distinguishing it from HA binding. In contrast to the N6 and N9, both sialidase and receptor-binding functions reside in the N2 active site, yet the catalytic and receptor-binding sites do not appear to be identical since relative sensitivity to inhibition of the two functions varies with oseltamivir, zanamivir, and peramivir (Mohr et al., 2015). Substitution of aspartate at position 151 near the active site to glycine, alanine or asparagine, or threonine 148 to isoleucine in H3N2 NAs (Lin et al., 2010; Mohr et al., 2015) or glycine 147 to arginine in N1 NAs (Hooper and Bloom, 2013) correlates with the acquisition of receptor binding. An H150R substitution has also recently been shown to correlate with NA receptor binding and has been found in both clinical samples and passaged viruses (Mogling et al., 2017). Interestingly, the affinity of the NA receptor-binding site to sialyl lactose is much stronger than the corresponding affinities of HA with its sialylated receptors (Zhu et al., 2012). The fact that entry and infection of MDCK cells with viruses having NA D151G can be blocked by NAIs (Gulati et al., 2013) suggests this NA active site-associated receptor binding function may play an important biological role for these H3N2 isolates.

### Virus Internalization

NAIs were found to reduce infection efficiency of cell lines without inhibiting virus binding or fusion activity, supporting a role for the NA during the viral entry process (Ohuchi et al., 2006). It was proposed that the NA facilitated movement of the virus across the cell surface by repeated binding and release steps from an endocytosis inactive site on the cell, to an active site, thereby increasing the efficiency of viral uptake. With the recent development of a biolayer interferometry assay, there is now clear evidence that viral NA plays a major role in driving virus particles over sialylated receptor surfaces. Using this method, Guo et al. (2018) showed that NA contributed to the initial rate of virus binding to sialoglycoproteins after which multiple low-affinity HA-sialic acid interactions take place. The rapid association and dissociation of these allow the NA to remove sialic acids and create a receptor density gradient that enables the rolling of virus particles across the surface.

NA has also been shown to enhance HA-dependent influenza virus fusion and infectivity using a cell-cell fusion assay and an HIV-based pseudotype infectivity assay (Su et al., 2009). When the NA gene from H9N2, H5N1, or A(H1N1)pdm09 virus was expressed on a PR8 background, the replication kinetics were similar *in vitro* (MDCK cells) and *in vivo* (mice) (Chen et al., 2013), yet the initial infection kinetics and virus-induced fusion and elution from erythrocytes were affected, implicating a role for NA during the early stage of infection (Chen et al., 2013).

### Catalytic Activity

By far, the most characterized function of NA is its action as a sialidase enzyme, enabling release of new virion progeny by enzymatically cleaving sialic acids from cell surface receptors and from carbohydrate side chains on nascent virions (Gottschalk, 1958; Palese et al., 1974). When NA activity is inhibited by the use of antivirals that target the enzymatic site, or through alteration of key amino acid residues, such as those identified to be integral in the catalytic process, the budding virions aggregate on the cell surface instead of being released (Lentz et al., 1987; Tarbet et al., 2014; Yang et al., 2016). This clumping of virions is due to HA on newly released virus binding to the sialic acids expressed on receptors in the vicinity of the budding site and to carbohydrate side chains on the HA and NA of progeny viruses that still contain terminal sialic acids in the absence of NA activity.

The catalytic mechanism of NA has not yet been completely resolved but is expected to begin with the binding of substrate to the active site *via* interactions with the catalytic residues and involves salt-bridge formation between the carboxylate of the sialic acid and the three arginine cluster at one end of the active site. Functional and structural evidence for the formation of a covalent intermediate between the C-2 on the sugar ring and the Tyr406 was obtained using a 2,3-difluoro sialic acid derivative (DFSA), which exhibits slow turnover, permitting accumulation of the covalent intermediate (Kim et al., 2013; Vavricka et al., 2013). This confirmed that the Tyr406 functions as the catalytic nucleophile. This leads to a change in the chair conformation of sialic acid with the carboxylate in the axial position, to a boat conformation with the carboxylate rotated into the pseudo equatorial position, then eventual cleavage of the sialic acid molecule from the preceding galactose residue. This cleavage first results in the release of sialic acid in an α-anomer conformation, then conversion into a β-anomer state shortly thereafter (Air, 2012).

The optimal activity of NA occurs at a pH range of 5.5–6.5 (Mountford et al., 1982; Lentz et al., 1987; McKimm-Breschkin et al., 2013); however, some viruses have been reported to have a stable NA activity at a lower pH range of 4–5, which has been shown to enhance replication kinetics (Takahashi and Suzuki, 2015). The presence of Ca^2+^ is thought to be essential during the reaction for both thermostability and enzyme activity of the NA. Using common fluorometric activity assays, increasing calcium ion concentration was shown to increase NA activity (Dimmock, 1971; Potier et al., 1979; Chong et al., 1991; Johansson and Brett, 2003). In crystal structures of NA bound to sialic acid, up to 5 Ca^2+^ ions per subunit that forms each tetramer are observed (Varghese et al., 1983; Russell et al., 2006; Xu et al., 2008; Lawrenz et al., 2010). An X-ray crystal structure of a whale N9 NA revealed that there were structural alterations near the substrate-binding site in the absence of calcium (Smith et al., 2006).

### NA Substrate Specificity

Avian IAVs express HAs that typically bind to galactose in an α2-3 linkage (SAα2-3Gal). For avian influenza viruses to undergo human to human transmission, the HA must acquire the capacity to bind SAα2-6Gal through mutations within the receptor-binding pocket (Gambaryan and Matrosovich, 2015). In some instances, the mutations are such that the HA will retain SAα2-3Gal binding and have dual specificity. Through further evolution in humans, the HA can become solely specific for SAα2-6Gal (Couceiro et al., 1993; Matrosovich et al., 1997, 2007). Similar to HA, the specificity of the active site of the viral NA evolves with time in the human host toward SAα2-6Gal (Kobasa et al., 1999; Gambaryan and Matrosovich, 2015). However, unlike the HA, it always maintains the ability to cleave SAα2-3Gal, even in viruses whose sole HA specificity is for SAα2-6Gal (Baum and Paulson, 1991; Kobasa et al., 1999). This evolution of specificity for sialic acid bound to galactose in different conformations is most likely due to the presentation of these glycoproteins on the surface of the target epithelium. In the upper airways, human tracheal epithelium expresses sialylated glycoproteins that are bound in an SAα2-6Gal linkage (Couceiro et al., 1993). In contrast, human bronchial mucus secretions contain large glycoproteins that express SAα2-3Gal, and as such, maintenance of NA activity for this linkage may be necessary for virion movement through the mucus barrier.

Functional evolution of NA has been shown to occur by amino acid substitutions that subtly alter the conformation of the NA catalytic domain to enable a different form of sialic acid to bind to the active site (Kobasa et al., 1999). A single change of isoleucine 275 to valine in N2 NA enables the shift in NA specificity toward increased activity for SAα2-6Gal, while other mutations are thought to subtly alter the conformation of the active site to accommodate this linkage of sialic acid (Kobasa et al., 1999).

## HA:NA Balance

**Figure 5:**
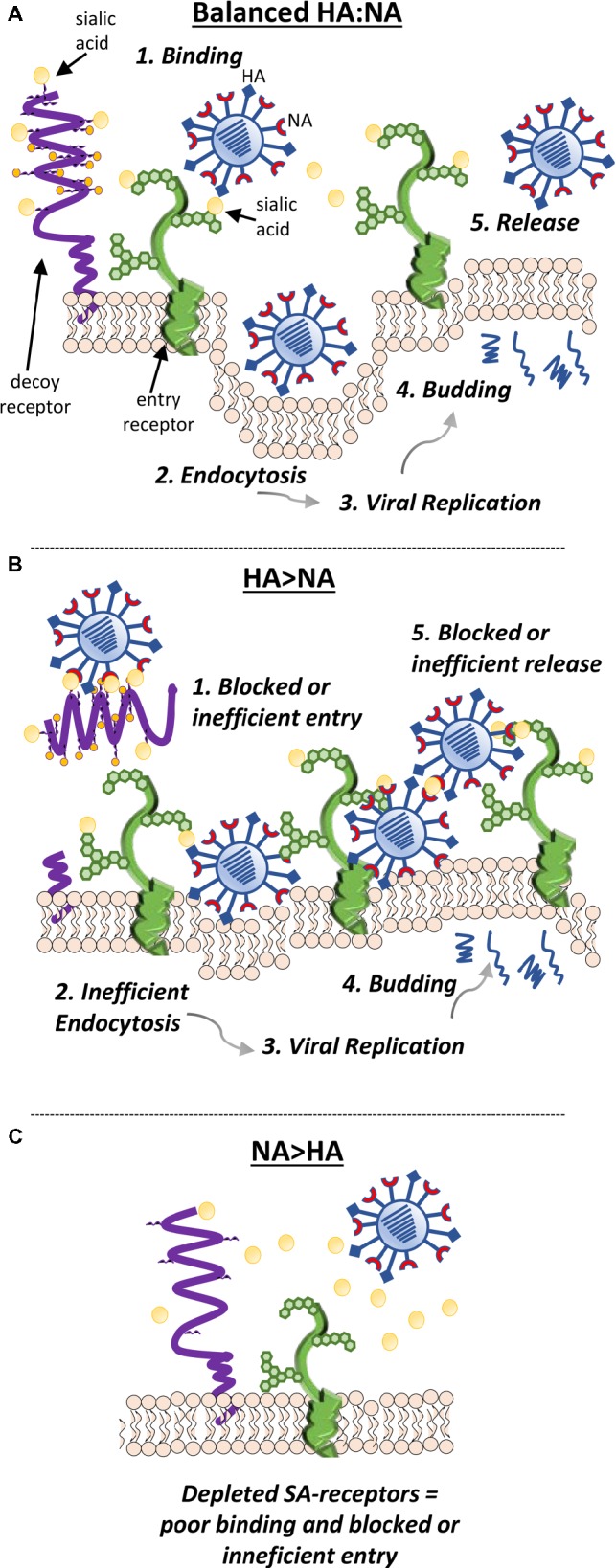
The relative activity of the HA and NA needs to be balanced to maintain the ability of the virion to efficiently infect and be released from cells. **(A)** Efficient cleavage of sialic acids from decoy receptors (such as cell-surface mucins) by NA enables HA access to sialic acids expressed by entry receptors and efficient endocytosis. After endosome escape of the viral genome and its migration to the nucleus, replication of the genome, synthesis of viral mRNAs, and translation of viral proteins take place. New virions assemble at the cell surface and are released from the cell by budding. As the viral components bud from the cell, NA cleaves sialic acids from receptors near the budding site to prevent virions binding back to the dying cell. NA cleavage of sialic acids from the carbohydrate side chains of nascent HA and NA also prevents newly budded virus from clumping together. Both these functions enable efficient release of the nascent virions from the cell. **(B)** If the HA and NA are mismatched and have mutations in important binding or catalytic sites that alter function, the relative activity of the two proteins may be imbalanced. If the sialidase function of NA is suboptimal, virus may remain bound by decoy receptors, which may shed and block virus entry into the cell. As the virus buds from the cell, an imbalance of HA and NA function may result in the lack of release of the virions due to the binding of HA to the sialic acids expressed at the cell surface that have not been removed by the NA. **(C)** Alternatively, if the sialidase activity of NA is too strong when compared to the HA-binding activity, sialic acids may be removed from receptors at the expense of the HA being able to bind and trigger endocytosis.

With respect to the ability for IAV to circumnavigate the mucosal environment and successfully infect underlying epithelial cells, the HA and NA need to have complementary receptor and ligand-binding specificity. It is also imperative that the relative activity of the two proteins is balanced to maintain the ability to infect and to release from cells efficiently (Figure 5). The importance of this functional balance was initially demonstrated when the first NA inhibitor resistant mutants were analyzed. Unexpectedly, rather than mutations in the NA, these drug resistant viruses had mutations in the HA (McKimm-Breschkin et al., 1996a,b, 1998). The HA mutations were found to reduce the affinity for receptors, so that less NA activity was required for virion release. However, while they had a fitness advantage in the presence of NA inhibitors, in the absence of NA inhibitors, the receptor binding was so poor for some of these mutants that the NA was able to cleave off the receptors before HA binding could take place. Such viruses are thus drug dependent (McKimm-Breschkin et al., 1996a; Blick et al., 1998). Others subsequently confirmed the need for balanced HA and NA activities (Wagner et al., 2002). The relevance to *in vivo* adaptation of influenza virus was shown by the isolation of several H3N2 viruses from patients that were reported to have little or no NA activity (Ferraris et al., 2006). A weak-binding HA was found to compensate for the absence of NA activity (Richard et al., 2012). Obviously, evolution of an HA or NA that negatively impacts viral attachment, replication, and transmission results in a less fit virus. In order to survive, compensatory mutations are needed to restore fitness (Lin et al., 2010; Mohr et al., 2015). Thus, for a human virus to gain efficient access to the cell surface *in vivo*, it needs to have a combination of HA and NA activities that enable escape from inhibition in the mucus layer by having either an HA with low avidity for mucin-bound SAα2-3Gal or an NA with greater activity for SAα2-3Gal, or a combination of the two. To attach and enter a cell, HA avidity for SAα2-6Gal must be strong enough to enable binding before the NA can cleave receptors. However, HA binding cannot be too strong, since release of progeny virions and prevention of aggregation at the cell surface need access of the NA to cleave the SAα2-6Gal.

Traditionally, incubation of IAV with red blood cells at different temperatures enables the functional balance of the HA and NA to be explored. As NA is inactive at 4°C but active at 37 °C, the ability of HA to agglutinate red cells at 4°C and of NA to elute virus at 37°C can be measured. However, this is no longer so clear cut. The isolation of the NA inhibitor-resistant HA mutants revealed that elution can occur rapidly with little NA activity, due to a weak HA (McKimm-Breschkin et al., 2012, 2013). More insight into the relative roles can be obtained by further modifications of the HA elution assay. When the eluted virus/red blood cells are mixed and re-incubated at 4 °C, if elution is due to a weak HA, virus will rebind. If the NA has cleaved the receptors, virus cannot rebind as in the traditional reaction. Alternatively, the addition of NA inhibitors will prevent elution if it is by the NA, but not by the HA (McKimm-Breschkin et al., 2012, 2013). Furthermore, NA inhibitors will also prevent agglutination if it is through the NA active site, as seen for the H3N2 viruses (Lin et al., 2010; Mohr et al., 2015). However, even with these variations on the assay, binding and elution vary depending on the species from which the red blood cells are derived from and are due to different sialic acid linkages presented at their surface (Ferraris et al., 2006; Richard et al., 2012).

Thus, the optimal functional balance of the HA and NA glycoproteins is difficult to measure as a number of physical characteristics of the virus can influence the properties of the HA- and NA-mediated receptor interactions. HA and NA affinity for and kinetics of interaction with sialic acids are the foremost determinants for the ability of the virion to infect a cell. On an average virion, there are 300–400 HA spikes and 40–50 NA spikes (Harris et al., 2006). The excess of HA over NA is perhaps reflective of the weak interaction of HA for sialic acids (Sauter et al., 1989) and the need to form several connections to form a stable interaction. The position and amount of NA present on the virion then plays an important role in gaining access to the cell surface sialic acids; its catalytic activity can directly influence efficiency of viral release, movement through mucus, and potential infection of new cells. Virion morphology can also contribute to altering the protein distribution and amount of NA and HA on the virion surface, potentially altering viral fitness and replication kinetics (Wasilewski et al., 2012). Finally, the ability for NA to access substrate either by the distribution of the NA on the virion or by variation in the length of protrusion of the NA spike can significantly influence both HA binding to a receptor and cleavage by NA from the receptor (Els et al., 1985; Castrucci and Kawaoka, 1993; Baigent and McCauley, 2001; Durrant et al., 2016). The recent study of Guo et al. (2018) further implicates the critical importance of HA:NA functional balance on virion movement through the mucus layer and over epithelial surfaces, a dynamic rolling process that may also be involved in cell-to-cell spread across the respiratory epithelium.

## Conclusion

Rather than just a sialidase that facilitates virus release from infected cells, the NA is a complicated multifunctional protein with an important role at many stages of the infectious process. While the NA is the main target for current antiviral therapies (Ison, 2015), recent approaches to new influenza therapy include targeting the HA with monoclonal antibodies (Nachbagauer and Krammer, 2017). However, given the NA also has the capacity to bind receptors, there needs to be caution in this approach, as it is possible that compensating mutations in the NA may allow escape from inhibition of the HA. As antibody levels against NA in children, adults, and the elderly correlate well with functional neuraminidase inhibition titers (Rajendran et al., 2017), altering vaccine strategies to enable efficient boosting of broadly cross-reactive antibodies against neuraminidase (Sandbulte et al., 2007; Marcelin et al., 2011; Liu et al., 2015; Chen et al., 2018) may be an important consideration in the campaign against the incredibly adaptable influenza virus.

## Author Contributions

JM and JM-B contributed to the conception and design of the review. JM wrote the first draft of the manuscript and designed figures. BG, ST, LB, and JM-B contributed to various sections of the manuscript, including figures. All authors contributed to manuscript revision, read, and approved the submitted version.

### Conflict of Interest Statement

The authors declare that the research was conducted in the absence of any commercial or financial relationships that could be construed as a potential conflict of interest.
